# Enhanced Cement Foam Composite with Biochar for Eriochrome Black T Dye Removal

**DOI:** 10.3390/ma18051158

**Published:** 2025-03-05

**Authors:** Mohammed Ettahar Boussalah, Malika Medjahdi, Sofiane Guella, Dominique Baillis

**Affiliations:** 1 GCE Laboratory, Djillali Liabes University, Sidi Bel Abbes 22000, Algeria; zakiboussalah22@gmail.com; 2 APELEC Laboratory, Sidi Bel Abbes 22000, Algeria; 3 LGPME Laboratory, Sidi Bel Abbes 22000, Algeria; gelsof@yahoo.fr; 4 LaMCoS, INSA-Lyon, CNRS UMR5259, 69621 Villeurbanne, France; dominique.baillis@insa-lyon.fr

**Keywords:** cement foam composite, biochar, wastewater, Eriochrome Black T, adsorption

## Abstract

Cement-based foam composites have gained attention as innovative and high-performing adsorbents for wastewater treatment due to their lightweight, porous, and structurally robust properties. This study investigates the adsorption of Eriochrome Black T dye onto biochar-modified cement foam, providing a cost-effective solution for industrial wastewater management. The integration of biochar into cement foam enhances its surface area and adsorption capabilities while maintaining structural stability and tunable porosity. The composites were characterized using scanning electron microscopy, Fourier-transform infrared spectroscopy, and energy-dispersive X-ray spectroscopy to verify quality and functionality. The adsorption process adhered to the Freundlich isotherm model (R^2^ = 0.967), indicating multilayer adsorption, with a maximum capacity of 13.33 mg/g under optimal conditions. Kinetic studies showed a pseudo-first-order fit (R^2^ = 0.981), while thermodynamic analysis revealed a spontaneous and endothermic process, with ΔH° = 28.84 KJ/mol and ΔG° values ranging from −0.457 to −2.36 KJ/mol. These results demonstrate the composite’s exceptional efficiency and scalability, making it a sustainable and practical option for removing persistent dyes such as Eriochrome Black T. This work contributes significantly to the advancement of environmentally friendly wastewater treatment technologies.

## 1. Introduction

The increasing presence of synthetic dyes in industrial wastewater has raised serious environmental and public health concerns [1,2]. Dyes are widely used across various industries, including textiles, leather, paper, and food, leading to the discharge of large volumes of colored wastewater [3,4,5]. Among these dyes, azo dye [6], which accounts for more than half of all synthetic dyes produced, are particularly problematic. Eriochrome Black T (EBT) [7,8], a sulfonated azo dye, is commonly utilized in textile dyeing and as an analytical reagent. Due to its high water solubility, stability, and resistance to biological degradation, EBT persists in water bodies, posing toxic and potentially carcinogenic effects on aquatic life and humans. Thus, removing EBT from wastewater before its release into the environment is critical [8].

Conventional methods for dye removal [9,10,11,12,13], such as coagulation [14,15,16,17,18], flocculation [17,18], oxidation [19,20], and membrane filtration [21,22], have demonstrated limited success. These techniques often involve high operational costs, complex chemical treatments, and sometimes generate secondary pollutants [23]. As a result, adsorption has emerged as a viable and more sustainable alternative for dye removal due to its effectiveness, simplicity, and cost-efficiency [24]. Among adsorbent materials, activated carbon (AC) is recognized for its high surface area and exceptional adsorption properties. However, the relatively high production cost of AC can restrict its practical application on a larger industrial scale [25,26].

To address this, researchers have focused on developing composite adsorbents for wastewater treatment that combine activated carbon with inexpensive, stable materials, making them more suitable for widespread use [27,28]. In this study, we investigate a cement-activated carbon composite (CACC) as an adsorbent material for removing EBT from wastewater. Cement foam serves as a low-cost [29], structurally stable matrix, while activated carbon, derived from agricultural waste, enhances the adsorption capacity of the composite [1]. This combination leverages the stability of cement and the adsorptive efficiency of activated carbon, offering a potentially economical solution for dye removal.

Beyond its role in adsorption processes, foam concrete—a lightweight material with densities typically below 200 kg/m^3^—is gaining attention for its environmental applications and innovative modifications. Previous studies have demonstrated how integrating various additives can enhance foam concrete’s structural, mechanical, and functional properties. For instance, the inclusion of microsilica nanoparticles, polypropylene fibers, and carboxymethyl cellulose (CMC) has significantly improved the compressive strength, pore structure uniformity, and foam stability of concrete [30,31]. Furthermore, the integration of industrial by-products, such as iron tailings, into cement-based matrices has showcased the material’s potential for sustainable construction and waste utilization [32]. Foam composites, such as polymeric foam-based polyurethane composites and concrete, provide a sustainable solution for wastewater and contaminated water treatment [31,33,34,35,36,37,38,39,40].

This study builds upon these advancements by combining the adsorption capabilities of activated carbon with the structural benefits of cement foam. The purpose is to analyze the adsorption behavior of EBT onto CACC through isotherm, kinetic, and thermodynamic modeling. We aim to characterize the adsorption process using Langmuir, Freundlich, Temkin, and Elovich models while evaluating adsorption kinetics with pseudo-first-order, pseudo-second-order, and Elovich models. Additionally, the thermodynamic parameters are calculated to assess the spontaneity, heat effect, and entropy changes [7,40,41]. These insights not only contribute to wastewater treatment research but also highlight the versatility of modified foam concrete for broader environmental and industrial applications.

## 2. Materials and Methods

### 2.1. Materials

**Figure 1:**
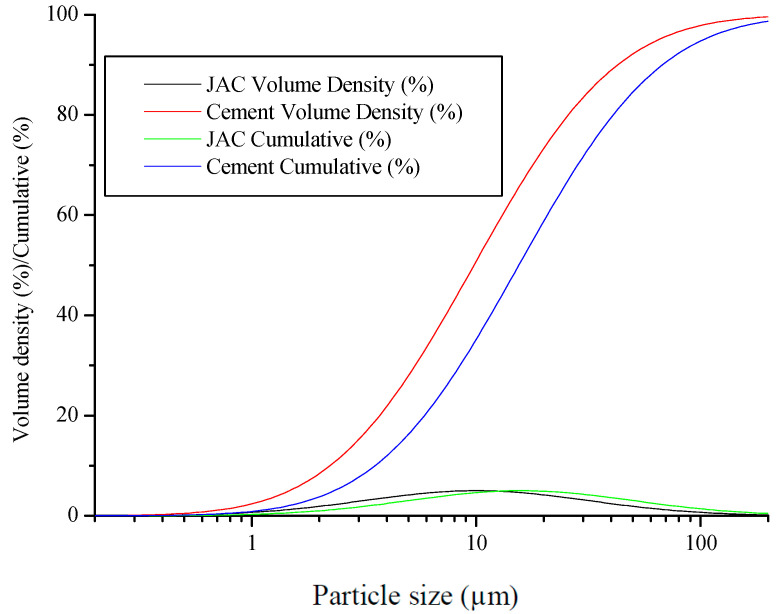
Particle size distributions of JAC biochar and cement.

**Figure 2:**
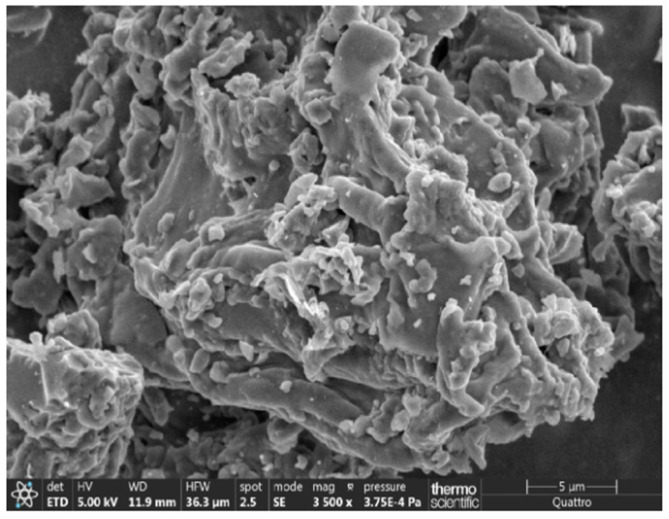
SEM image of JAC biochar.

The cement foam-activated carbon composite (CACC) was synthesized by blending activated agricultural waste-derived activated carbon (jujube core-activated carbon (JAC) with BET surface area = 408.224 m^2^/g in proportions of 0%, 5%, 10%, and 15%) with cement (provided by GICA group-Algeria, CEM II/ B-L 32.5 N NA 442), water, and a foaming agent. The jujube core-activated carbon (JAC) was prepared using H_3_PO_4_ activation and pyrolysis at 600 °C for 2 h in the laboratory with jujube cores (Djelfa, Algeria). The particle size distribution of JAC biochar and cement is shown in Figure 1. JAC biochar exhibits a particle size range predominantly between 1 µm and 100 µm, with a peak volume density occurring within the 1–10 µm range. For cement, the particle size range is slightly broader, predominantly between 1 µm and 100 µm, with a peak in the 10–30 µm range [42]. The cumulative distribution curve indicates that the D50 value, representing the median particle size at which 50% of the particles are smaller, is approximately 9 µm for JAC biochar and 21 µm for cement. These results were obtained using a laser diffraction analyzer (Mastersizer 3000, Malvern Panalyticalm Malvern, UK). The porous structure of the JAC biochar powder was examined through scanning electron microscopy (SEM series ZEISS EVO, Zeiss, Oberkochen, Germany), as depicted in Figure 2. The chemical compositions of the JAC biochar was measured with an X-ray fluorescence analyzer, and the results are shown in Table 1. A foaming agent based on anionic surfactants was used with a dilution ratio of 30–40. In this study, the composition details of cement foam-activated carbon composite (CACC) are provided in Table 2.

### 2.2. Characterization

We aimed to investigate the effect of jujube core-activated carbon (JAC) on the cement foam matrix, specifically its influence on carbonation processes while preserving the material’s intrinsic chemical properties. The chemical composition of the material was determined using energy-dispersive X-ray spectroscopy (EDX) with a ZEISS EVO SEM equipped with an integrated analyzer. Furthermore, Fourier-transform infrared spectroscopy (FTIR) was conducted using a Nicolet iS 10 (Thermo Fisher Scientific, Waltham, MA, USA) spectrophotometer equipped with a macro-ATR (attenuated total reflectance)

## 3. Experimental Procedures

### 3.1. Mix Proportions of Foamed Cement and JAC-Modified Foam (CACC)

**Figure 3:**
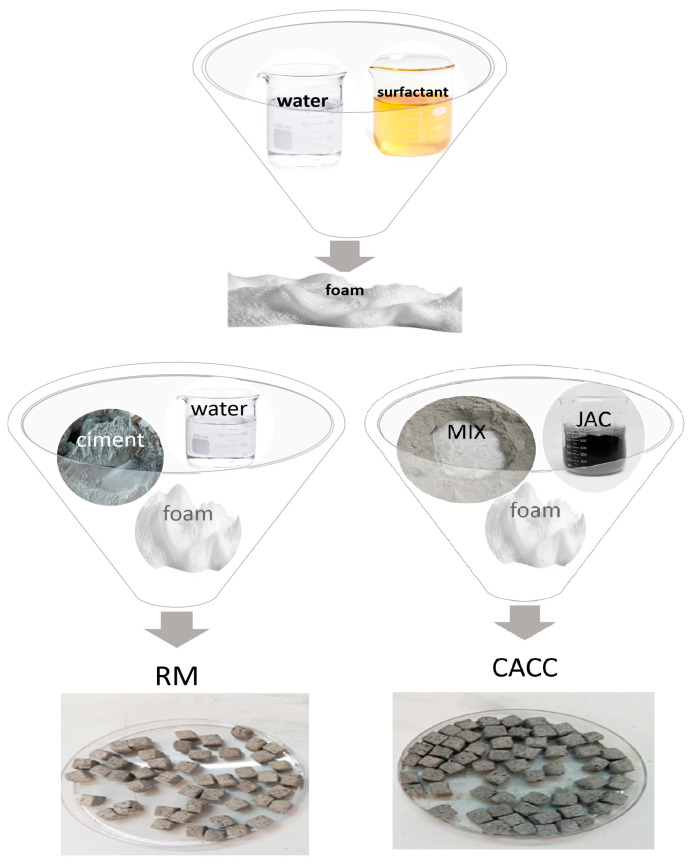
Preparation steps of RM and CACC samples for characterization and application.

As shown in Table 2, foamed cement mixtures with varying JAC dosages were prepared. To ensure stability and workability [1,43], a consistent water-to-binder (w/b) ratio of 0.5 was maintained, along with a fixed foam ratio of 12.5%. The mixtures included JAC dosages of 0% (RM), 5% (CACC5), and 15% (CACC15) relative to the binder. Figure 3 depicts the preparation steps of the RM and CACC samples.

### 3.2. Adsorption Experiments

**Figure 4:**
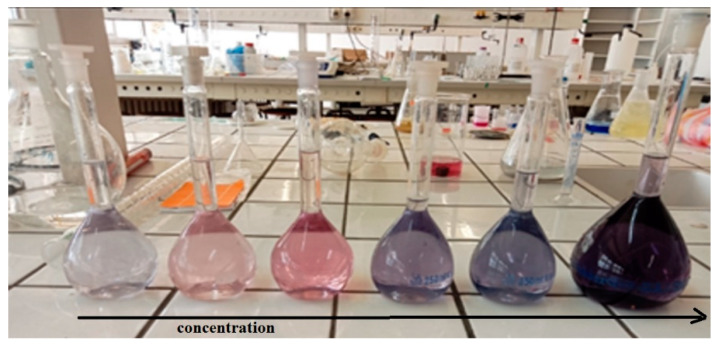
EBT solutions with different concentrations.

**Figure 5:**
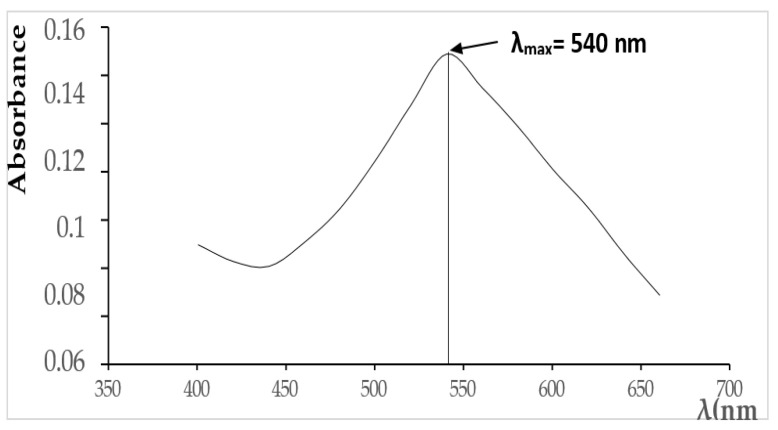
UV absorption spectrum of Eriochrome Black T (EBT).

Adsorption experiments were carried out in batch mode using varying concentrations of EBT dye solutions. The solutions (Figure 4) were continuously stirred to ensure uniformity, and the equilibrium concentration of EBT was determined at 540 nm using spectrophotometry (Figure 5).

The adsorption capacity (qe, mg/g) of the adsorbent (RM, CACC15), which represents the amount of dye adsorbed per unit mass of adsorbent, was calculated using the following standard adsorption equation:

(1) $$q_{e\left(\frac{mg}{g}\right)=\frac{\left(C_{0}-C_{e}\right)V}{m}}$$

where *C*o and *C*e are the initial and equilibrium concentrations (mg/L), *m* is the sorbent mass (*g*), and *V* is the volume of the dye solution (L).

## 4. Experimental Results

### 4.1. Composition and Microstructure Characterization

**Figure 6:**
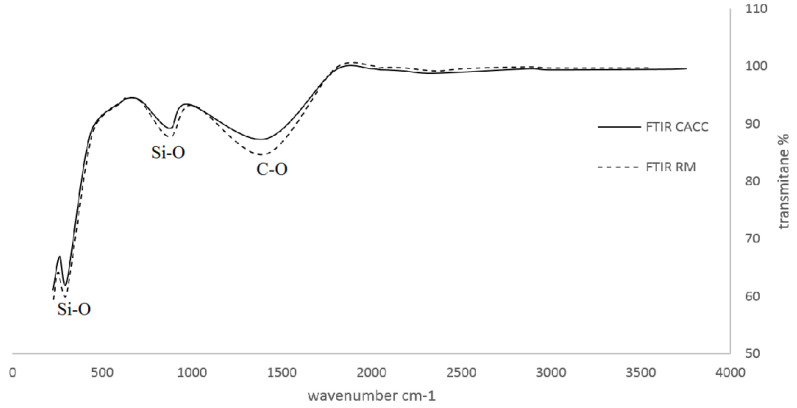
FTIR spectra of modified foam cement (CACC) and raw foam cement (RM).

**Figure 7:**
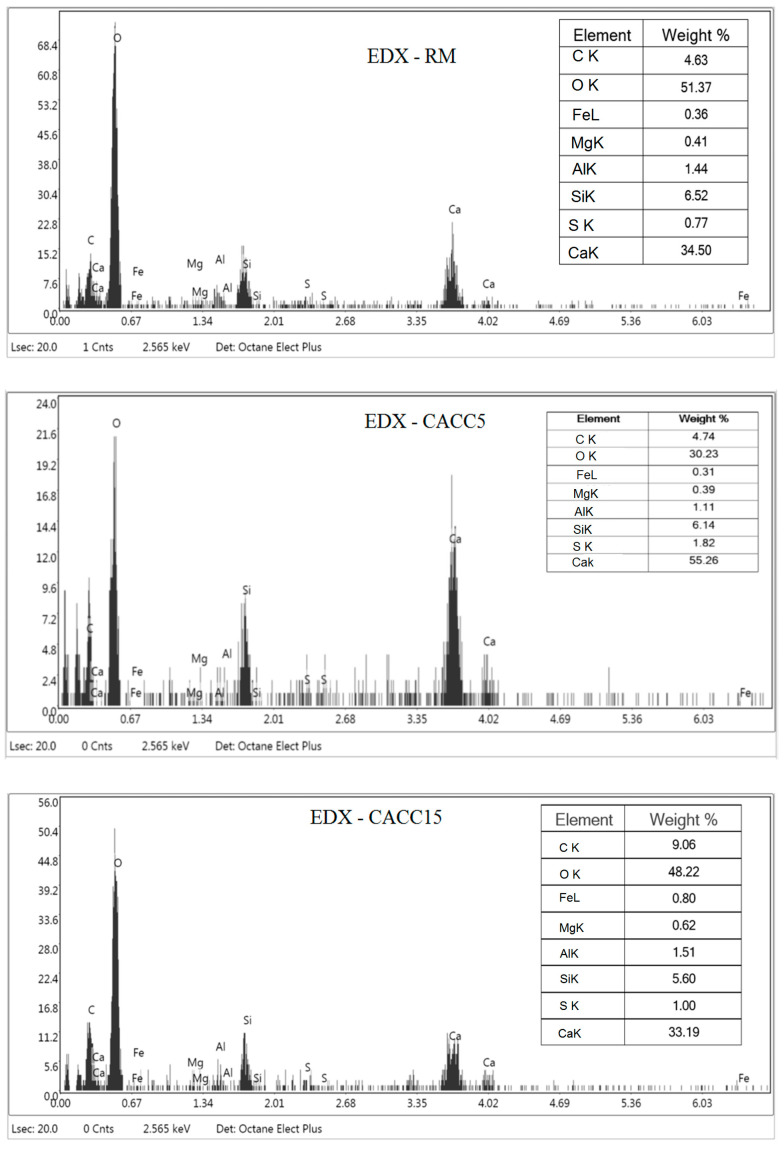
EDX spectra of foamed cement (RM) and modified foamed cement (CACC).

The analysis of the FTIR (Figure 6) and EDX (Figure 7) results demonstrates that the addition of jujube core-activated carbon (JAC) to the cement foam matrix positively influences its carbonation processes while maintaining the material’s fundamental chemical composition.

The FTIR spectrum reveals sharper and more intense peaks at 470 cm^−1^ and 970 cm^−1^ [44], corresponding to Si-O bending and Si-O stretching vibrations, respectively. These peaks are characteristic of calcium silicate hydrate (C-S-H), a critical hydration product in cement. The increase in their intensity (e.g., absorbance rising from 0.60 to 0.67 for the 470 cm^−1^ peak and from 0.72 to 0.80 for the 970 cm^−1^ peak) indicates enhanced hydration in the presence of activated carbon. This observation is corroborated by the EDX spectrum, where silicon (Si) is measured at 6.14 and 5.6 wt% in the CACC5 and CACC15 sample, respectively, compared to 6.52 wt% in the RM sample. Moreover, the results confirm that carbonation is significantly promoted in the CACC cement foam. The FTIR spectrum shows an intensified peak at 1410 cm^−1^, associated with C-O stretching vibrations of carbonate species (CaCO₃) [45]. This peak’s increased absorbance (from 0.76 to 0.84) suggests greater carbonate formation in the presence of activated carbon. The EDX spectrum supports this observation, with the carbon (C) content rising from 4.63 wt% in the RM sample to 4.74 and 9.06 wt% in the CACC5 and CACC15 samples, respectively. This increase in carbon content reflects the biochar’s integration into the matrix and its role in enhancing carbonation. The interaction between the carbon-rich biochar and calcium ions leads to the formation of stable carbonate phases, which contribute to pore filling, densification of the matrix, and improved durability.

### 4.2. Adsorption Studies

The adsorption process of Eriochrome Black T (EBT) dye using cement-activated carbon composite (CACC) was investigated through a series of kinetic experiments. CACC15 (15% JAC 200 µm) was selected due to its high porosity and the efficiency of activated carbon in adsorbing dyes from aqueous solutions.

#### 4.2.1. Adsorption Isotherms

**Figure 8:**
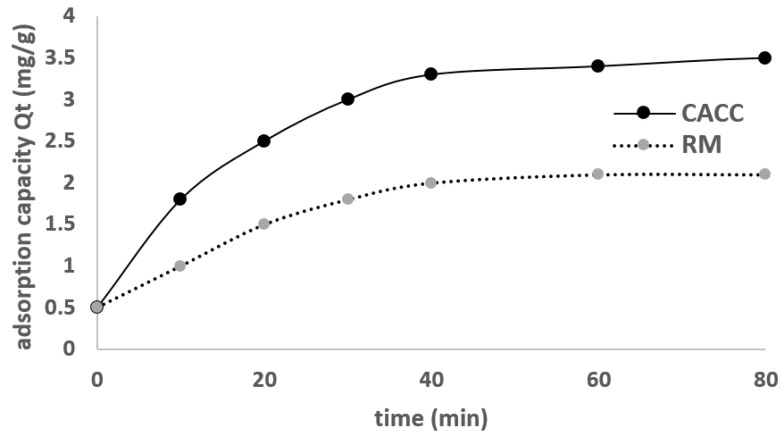
EBT dye kinetic adsorption by CACC and RM (T = 29 ± 1 °C, adsorbent dose 0.3 g CACC15, C0 = 20 mg/L, stirring speed = 0 rpm).

**Figure 9:**
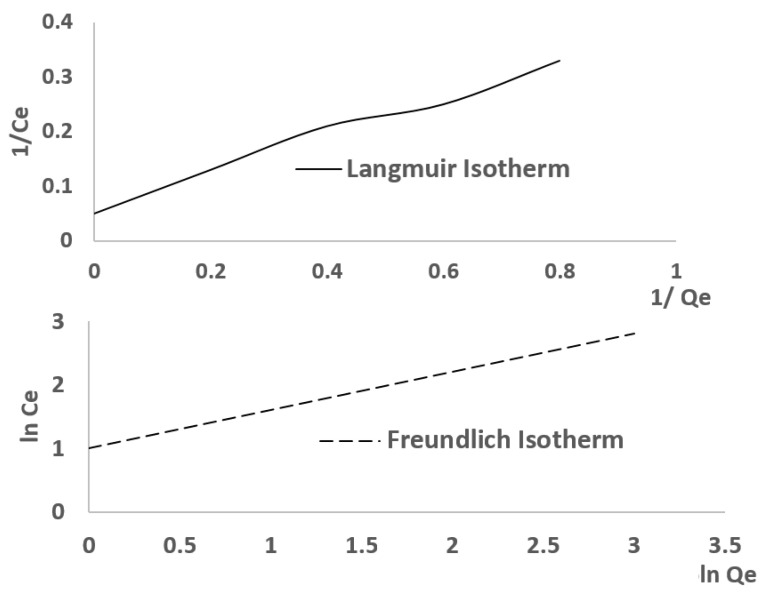
Langmuir and Freundlich isotherm for adsorption of EBT on CACC.

Regarding Figure 8, the adsorption behavior of CACC likely follows the Langmuir isotherm due to its rapid saturation and higher adsorption capacity; however, the Freundlich model provides a better fit (Table 3), indicating slight surface heterogeneity. For RM, the Freundlich isotherm is more appropriate, as its lower adsorption capacity and slower kinetics align with adsorption on a heterogeneous surface. While both materials exhibit some Langmuir behavior, as evidenced by the plateaus in the kinetic curve, the overall isotherm model fitting suggests that the Freundlich isotherm is the dominant model for both materials, particularly for RM. Figure 9 depicts the Langmuir and Freundlich isotherm for the adsorption of EBT on CACC.

#### 4.2.2. Kinetic Modeling

**Figure 10:**
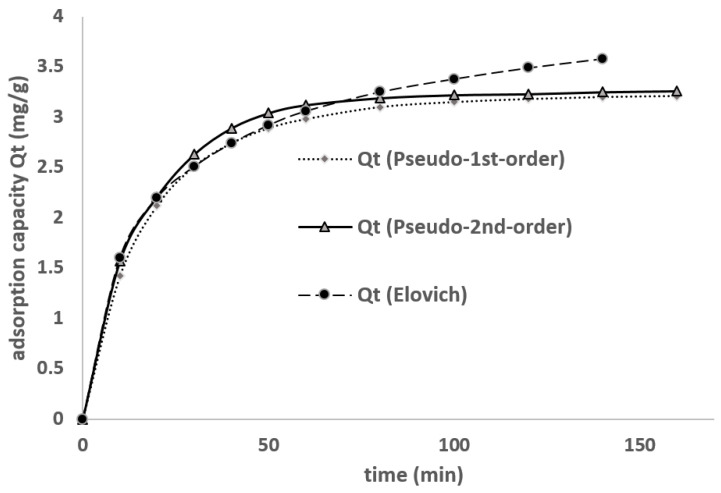
Kinetic analysis modelling.

The pseudo-first-order, pseudo-second-order, and Elovich kinetic models (Figure 10) were evaluated, and the summary of kinetic models for EBT adsorption on CACC is shown in Table 4.

The kinetic analysis of EBT adsorption onto CACC was conducted using three models: pseudo-first-order, pseudo-second-order, and Elovich models. The results, summarized in Table 1, demonstrate that the pseudo-first-order model provides the best fit, with an R^2^ value of 0.981 and a close agreement between the calculated sorption capacity (qe,cal = 3.206 mg/g) and the experimental sorption capacity (qe,exp = 3.14 mg/g). The pseudo-second-order model, with an R^2^ value of 0.943, and the Elovich model, with an R^2^ value of 0.881, display less accurate fits. These findings confirm that physical adsorption, driven by weak Van der Waals interactions, is the primary mechanism governing the adsorption process.

#### 4.2.3. Thermodynamic Analysis

Because of the decrease in solution viscosity, it is known that an increase in temperature accelerates the diffusion of adsorbate molecules through the outer boundary layer and into the adsorbent particles [47,48].

**Figure 11:**
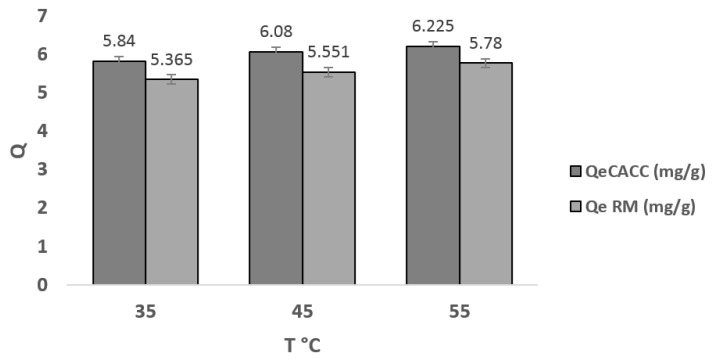
Temperature effect of EBT dye adsorption on CACC15 (t = 150 min, C0 = 40 mg/L).

From Figure 11, we observe that the temperature is a kinetic factor favoring the adsorption of the dye and also that the quantity adsorbed increases from 5.84 to 6.225 mg/g for CACC and from 5.365 to 5.78 mg/g for the raw material when the temperature increases from 35 °C to 55 °C. This increase is due to the effect of vibration of the dye molecules with the increase in shock numbers on the surface of the adsorbents. Therefore, the adsorption of the examined ions seems to be an endothermic phenomenon. This could also be due to a relative increase in the mobility of ions in solution, which improves their exposure to active adsorption sites on the one hand and makes it difficult for them to access sites on the other hand.

To calculate the thermodynamic parameters—free energy ΔG°, enthalpy ΔH°, and entropy ΔS°—we use the following equation [47]:

(2) $$log\frac{Qe}{Ce}=\frac{\Delta S{}^{\circ}}{2.303R}+\frac{-\Delta H{}^{\circ}}{2.303RT}$$

where ΔH° is enthalpy (kJ/mol), ΔS° is entropy in (J/mol. K), T is temperature in kelvin R is a perfect gas constant (R = 8.314 J/ mol K), *Qe* is the adsorbed quantity (mg/g), and *Ce* is the equilibrium concentration (mg/L).

**Figure 12:**
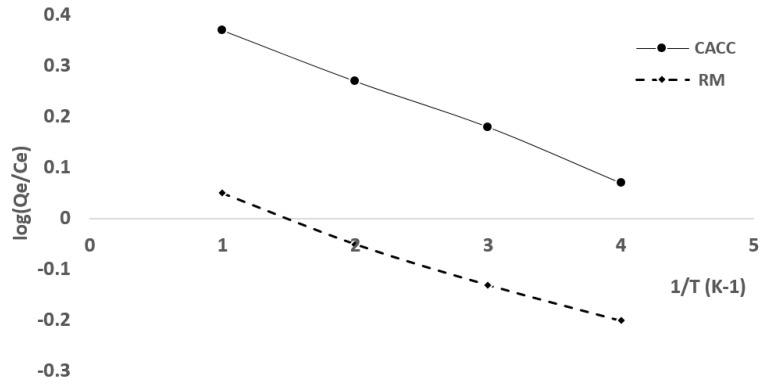
Representation of Van’t Hoff equation for EBT adsorption on CACC15 and on RM.

From the graph log (*Qe*/*Ce*) = f (1/T) in Figure 12, we find a straight line with a sloping ΔH°/*2.303R* and a y-intercept ΔS°/*2.303R*.

The results of the thermodynamic parameters are shown in Table 5.

From these results, it can be seen that the free energy (ΔG°) is negative in all cases except at low temperatures for RM. At 35 °C, the ΔG° for CACC is −0.457 kJ/mol, indicating that adsorption is spontaneous. However, for RM, the ΔG° is 1.008 kJ/mol, indicating non-spontaneity at this temperature. As the temperature increases, ΔG° becomes negative for both materials; at 45 °C, ΔG° for CACC is −1.409 kJ/mol, and for RM, it is 0.415 kJ/mol, becoming more negative at 55 °C where ΔG° for CACC reaches −2.36 kJ/mol, and for RM, it is −0.177 kJ/mol, suggesting that the process is spontaneous at higher temperatures. This indicates that EBT adsorption on both CACC and RM at 55 °C is spontaneous [49].

Regardless of temperature, the EBT adsorption process on CACC and RM is physisorption, as ΔG° values are below 20 kJ/mol for both materials, confirming the weak, non-chemical nature of the adsorption.

The enthalpy (ΔH°) is positive for both CACC (28.84 kJ/mol at 35 °C) and RM (19.27 kJ/mol at 35 °C), implying that the adsorption process is endothermic. This means higher temperatures facilitate adsorption, as more heat is required for the process to occur.

Additionally, the entropy (ΔS°) is positive for both materials—95.15 J/mol·K for CACC and 59.29 J/mol·K for RM—suggesting that the EBT molecules remain more disordered at the solid/solution interface during the adsorption process. This further supports the physisorption nature of the process, where the molecules are less ordered as they adsorb onto the surface [49].

These values suggest that higher temperatures favor the adsorption of EBT onto CACC, enhancing the overall adsorption capacity and confirming that the process is both spontaneous and endothermic.

Several studies on EBT removal using bio-activated carbon [50,51] and composite materials [52] indicate significant variations in adsorption behavior based on thermodynamic parameters. ACSB LA FeO₃ (activated carbon from sugarcane bagasse modified with lanthanum ferrite) exhibits an improved adsorption capacity compared to unmodified ACSB [52], likely due to enhanced surface properties. Among the studied adsorbents, CACC demonstrates the highest spontaneity and entropy change, indicating strong adsorption and a highly disordered system, making it a promising reusable material. In contrast, RM is non-spontaneous at 303 K, proving ineffective for EBT dye removal.

## 5. Conclusions

This study confirms that cement-activated carbon composite (CACC) is a highly effective and sustainable adsorbent for removing Eriochrome Black T (EBT) dye from wastewater, offering a practical solution for industrial applications. By combining the adsorptive efficiency of activated carbon with the durability and cost-effectiveness of cement, CACC emerges as an environmentally friendly option for dye-laden effluent treatment. Modeling adsorption isotherms, kinetics, and thermodynamics provides valuable insights into the adsorption mechanism. The Freundlich isotherm model (R^2^ = 0.967) best describes the process, suggesting multilayer adsorption on heterogeneous surfaces due to the composite having varied surface properties, where different adsorption sites exhibit distinct affinities for dye molecules. The maximum adsorption capacity (qmax = 13.33 mg/g) highlights CACC’s effectiveness compared to raw cement foam and many conventional adsorbents. Kinetic modeling further supports this finding, as the adsorption process aligns with the pseudo-first-order model (R^2^ = 0.981), indicating that physical adsorption is the primary mechanism, driven by Van der Waals forces rather than chemical bonding. Thermodynamic analysis reveals that the adsorption is spontaneous and endothermic, with negative Gibbs free energy (ΔG°) values decreasing from −0.457 kJ/mol at 35 °C to −2.36 kJ/mol at 55 °C, confirming spontaneity and a positive enthalpy (ΔH°= 28.84 kJ/mol) and indicating that higher temperatures enhance dye uptake. Additionally, the positive entropy (ΔS°= 95.15 J/mol·K) reflects increased randomness at the solid–liquid interface, suggesting favorable interactions between CACC and EBT molecules. Together, these findings validate CACC’s efficiency as a sustainable adsorbent with a strong capacity for multilayer adsorption, making it suitable for large-scale wastewater treatment. Future research should focus on optimizing CACC’s regeneration and exploring its broader application potential in adsorbing a variety of industrial pollutants, thus reinforcing its role in promoting cleaner water resources and sustainable environmental practices.

## Figures and Tables

**Table 1:** Chemical compositions of JAC biochar using loss on ignition (LOI) method.

Materials	SiO₂	Al₂O₃	Fe₂O₃	CaO	MgO	SO₃	Na₂O	K₂O	P₂O₅	Carbon	LOI
**JAC Biochar**	19.17	0.04	0.05	0.23	0.10	0.14	0.05	1.19	2.50	78.74	80.00

**Table 2:** Mix proportion of samples (mass %).

Sample	Cement	JAC (%)	Water	Water/Binder Ratio	Foam Ratio
**RM (control)**	100	0%	50	0.5	12.5%
**CACC5 (5%)**	98	2%	50	0.5	12.5%
**CACC15 (15%)**	95	5%	50	0.5	12.5%

**Table 3:** Comparative summary of isotherm model parameters for EBT adsorption on CACC.

Isotherm Model	Equations	Parameter	Value
*Langmuir*	$q_{e}=\frac{K_{L}.b.C_{e}}{1+(K_{L}.C_{e})}$	K_L_ (L/mg)	0.259
		Q_max_ (mg/g)	13.33
		R^2^	0.942
*Freundlich*	$q_{e}=K_{F}.C_{e}^{\frac{1}{n}}$	K_F_ (mg/g)	2.66
		n	1.716
		R^2^	0.967

**Table 4:** Summary of kinetic models for EBT adsorption on CACC.

Model	Parameters	R^2^	Mechanism	
Pseudo-First-Order	Qe = 3.20608 k1 = 0.03925	0.98143	Physical adsorption (Van der Waals forces)	[23]
Pseudo-Second-Order	Qe = 3.83352 k2 = 0.01165	0.94321	Partial chemisorption	[46]
Elovich	α = 0.31541 β = 1.16099	0.88124	Dominantly physical adsorption	[23]

**Table 5:** The thermodynamic parameters ΔG°, ΔH°, and ΔS° for the adsorption of EBT on CACC and RM.

Thermodynamic Parameters	T (C°)	ΔG° (KJ. Mole^−1^)	ΔH° (KJ. mole^−1^)	ΔS° (J. mole^−1^K^−1^)
*CACC*	35	−0.457	28.84	95.15
	45	−1.409		
	55	−2.36		
*Raw material*	35	1.008	19.27	59.29
	45	0.415		
	55	−0.177		

## Data Availability

Data is contained within the article.
